# Ketamine Cystitis Following Ketamine Therapy for Treatment Resistant Depression – Case Report

**DOI:** 10.1192/bjo.2025.10711

**Published:** 2025-06-20

**Authors:** Minna Chang, Allan Young, Mario Juruena

**Affiliations:** 1 Epsom & St Helier University Hospital Trust, London, United Kingdom; 2 King’s College and Maudsley, London, United Kingdom

## Abstract

**Aims:** Ketamine is a novel and exciting putative antidepressant medication for patients with treatment-resistant depression. A complication commonly seen in frequent and heavy recreational use of ketamine is ulcerative cystitis, which presents with lower urinary tract symptoms (LUTS) and upper renal tract damage and can be seen in over 25% of regular users.

Although ketamine-induced cystitis (KIC) is a recognised complication in recreational use of ketamine, its occurrence in therapeutic use of ketamine in depression has so far not been reported. The exact pathogenesis of KIC is currently unknown, making treatment and prevention advice much more difficult. Early diagnosis of KIC and immediate cessation of ketamine has been shown to improve adverse urinary tract symptoms and prevent further damage.

**Methods:** We present a case of a 28-year-old female who was started on ketamine treatment for depression, and who then developed symptoms of KIC, which was confirmed by urine microscopy, culture and analysis.

**Results:** Ketamine-induced cystitis (KIC) typically starts with urinary symptoms that include dysuria, urgency, nocturia and urinary frequency. With continued use, symptoms can progress to incontinence, haematuria, bladder wall fibrosis and ulcerative cystitis. Ongoing use of the drug can lead to involvement of the upper renal tract, including hydronephrosis and chronic kidney failure. Physical examination and investigations may demonstration suprapubic pain, sterile pyuria and increased eosinophils within the bladder wall. The pathophysiological mechanisms of LUTS are not yet fully understood and further research to define therapeutic options would be useful.

Imaging of the bladder in severe cases may demonstrate a grossly constricted bladder with thickened walls. Cystoscopy often demonstrates a friable bladder mucosa that is prone to bleeding. Microscopically, the urothelium may appear denuded, ulcerated and infiltrated by inflammatory cells, such as mast cells and eosinophils. Other findings include submucosal fibrosis, muscle hypertrophy and collagen deposition.

Although the exact pathogenesis of KIC is not yet fully understood, various mechanisms have been postulated and it is likely that several pathways are involved simultaneously.

One theory is that the ketamine and its metabolites (which are largely excreted by the urinary tract), cause direct toxicity to the bladder. These disrupt the urothelial integrity of the bladder epithelium and initiate interstitial fibrosis. This has been demonstrated in animal models and the level of damage directly correlates with the dose of ketamine used.

Another theory is an IgE-mediated response. Bladder samples in ketamine users frequently show raised inflammatory cells and messengers, including mast cells, eosinophils, COX-2 (cyclo-oxygynase-2), NOS (nitric oxide synthase) and IgE. These levels fall once the patient is in remission from ketamine use and rise again once ketamine is restarted. This suggests an inflammatory response or a hypersensitivity reaction leading to bladder damage.

Ketamine can also directly stimulate various chemicals, including adenosine triphosphate, antiproliferative factor and oxidative stressors, which subsequently lead to changes in the bladder wall. It has been reported that the NMDA receptor (NMDAR) and angiogenic factors can also cause microvascular injury within the bladder.

Other proposed theories include aberrant neurotrophic factors, protein kinase B, mTOR pathways, metadherin and MAPK pathways, leading to downstream fibrosis of the bladder.

Early diagnosis of KIC and immediate cessation of ketamine use has been shown to improve symptoms, reverse early disease and prevent further damage.

**Conclusion:** To our knowledge, this is the first reported case of KIC in a patient receiving treatment-dose ketamine as part of their antidepressant therapy.

